# Socio-spatial logic of demolition and relocation in urban China

**DOI:** 10.1371/journal.pone.0339631

**Published:** 2026-04-17

**Authors:** Zhe Lin, Gang Li

**Affiliations:** 1 Department of Geography, University of Colorado Boulder, Boulder, Colorado, United States of America; 2 Institute of Behavioral Science, University of Colorado Boulder, Boulder, Colorado, United States of America; 3 College of Urban and Environmental Sciences, Northwest University, Xi’an, Shaanxi, China; Hunan University, CHINA

## Abstract

While urban demolition (*Chai Qian*), a process encompassing housing removal and resident relocation, is a distinctive feature of China’s urban transformation, its underlying socio-spatial logic remains insufficiently understood. This paper extends Henri Lefebvre’s theory to examine the spatial patterns of demolition and their diverse driving routes. Using large-scale demolition data from five representative Chinese cities, a three-stage approach integrating spatial analysis, regression models, and text analysis identified a consistent “single hot core” pattern across all cities, shaped by network density, GDP, and population density. Road network and population density exerted location-specific influences, which indicates two dominant spatial logics: the renewal of dense, underdeveloped inner-city areas and the strategic preparation of well-connected zones for future development. GDP reinforced this dual logic. We proposed the Urban Demolition Complex Systems (UDCS) that conceptualizes demolition as a multi-scalar socio-spatial process shaped by interacting social, economic, and governance forces. This study provided a nuanced geographical perspective on the demolition process to illuminate how demolition operates as a spatially differentiated and policy-driven urban practice. In the context of rapid global urban transformation, our results help inform upstream, local government-level interventions to promote more equitable redevelopment strategies that support residents’ well-being and sustainable urban development.

## Introduction

In the process of urbanization, large-scale demolition and redevelopment have become the core driving forces behind urban spatial restructuring in developing countries worldwide [1]. China’s rapid urbanization over the past three decades has given rise to the world’s largest wave of urban renewal. More than 66,000 urban renewal projects have been implemented nationwide, with a total investment of 2.6 trillion yuan (approximately 364 billion US dollars) [2]. In China, this process is commonly known as “*Chai Qian*”, which refers to the demolition of housing or neighborhoods and the relocation of affected residents. It is closely tied to China’s unique land use and ownership policies. Chinese land is either state-owned (mainly in urban areas) or collectively owned (primarily in rural areas), which creates a distinct framework for urban expansion and redevelopment. For clarity and consistency, the term demolition is used throughout this paper to refer to “*Chai Qian*”.

The demolition process involves multiple stages: demolition, construction, relocation, and resettlement. Each stage entails the evolution of the human-land system, the reconstruction of space, and the reconfiguration of social networks. Demolition reasons gradually shifted from urbanization to gentrification [3]. Since the reform and opening-up policy, approximately 150–200 million people have been directly or indirectly affected by demolition [4]. While a limited body of research points to potential benefits of demolition for affected residents, particularly through substantial financial compensation in the form of lump-sum payments and replacement housing [5–7], the majority of studies emphasize its micro-level risks, including declines in household labor force participation [8], income reductions [9], and decreased investment in human capital [7].

Beyond these economic consequences, demolition and relocation processes can also impose significant psychosocial and mental health burdens. Displacement, uncertainty surrounding compensation, disruption of social networks, and the loss of place attachment are linked to heightened stress, anxiety, and emotional distress among affected residents. Like other social crises [10–12], demolition primarily affected lower-income citizens and older adults living in low-value properties [13,14]. They are often unable to afford the new housing that emerged in the area [15]. In the study on multi-stakeholder subjects, Zhang [16] investigated the micro-politics of urban village redevelopment in Jiaxiaoying Village in Hefei City and highlighted the complex interactions among the state, homeowners, tenants, and the elder generation during the demolition process. He found that the state employed an agent-based governance model and used neighborhood committees and clan networks to control grassroots society. Another study focusing on individuals in eastern Nanjing argued that rather than excluding peasants, demolition integrated rural spaces into the urban system by exploiting uneven urban-rural property regimes and considered demolition as an ongoing, hegemonic struggle over urban accumulation [17]. Some researchers viewed demolition as a necessary means of proactive urban industrial upgrading and structural adjustment. Between 2015 and 2019, Audin [18] studied unfinishedness and ruination in Datong after the construction boom by following the last remaining residents within an interrupted project. Stuck between “*Chai*” (demolition) and “*Qian*” (relocation), the study highlighted their urban experiences as city dwellers navigating a landscape of ruins and unfinished architecture.

Beyond individual- and household-level impacts, demolition also plays a critical role in shaping broader urban spatial structures. In rapidly urbanizing cities, redevelopment strategies that concentrate demolition and reinvestment in central areas can reinforce monocentric urban forms, which exacerbate spatial inequalities in access to employment, services, and amenities [19–21]. In contrast, recent planning scholarship has emphasized the potential of multicentric and spatially balanced urban configurations to promote more equitable access to daily needs, reduce commuting distances, and mitigate congestion and environmental pressures, a principle reflected in concepts such as “Isobenefit Urbanism” [22–24]. From this perspective, demolition sites are not only an outcome of urban restructuring but also strategic spatial opportunities through which cities may redistribute urban functions and reconfigure accessibility.

Existing studies have demonstrated the profound and long-lasting impacts of demolition on individuals’ life courses and urban development. However, this growing body of demolition research has largely overlooked demolition as a complex macro-spatial system. Key questions therefore remain unanswered: Is there a macro-scale spatial pattern of demolition? What factors drive these patterns? How do they vary within and across cities? This study examined five major Chinese cities: Shanghai, Nanjing, Hangzhou, Xi’an, and Chengdu based on multidimensional data. We investigated and compared spatial distributions and driving paths of demolition sites in five cities and situated demolition within a broader systemic framework that conceptualizes demolition as a key node that triggers the nonlinear evolution of urban systems. The findings have important implications for policymakers, helping them understand different paths of demolition across cities. More importantly, it provides a deeper understanding of the evolving human-environmental relationship, guiding the ongoing urbanization in Global South.

### Study area and data

**Fig 1:**
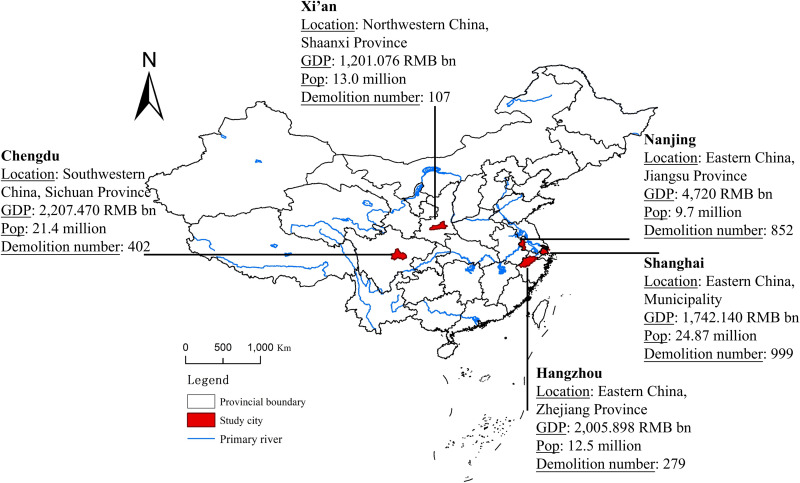
Geographical locations and key information of study cities.

To generalize the findings in Global South cities, we selected five representative cities with various developing paths. Shanghai, as the earliest coastal gateway, represents global cities in Global South focusing on global competitiveness and high-end services improvements. Nanjing, a historically significant political and cultural center in the Yangtze River Delta, represents regional center cities struggling to balance ecological protection and cultural heritage. Hangzhou, driven by the digital economy, exemplifies emerging digital cities advancing smart and green development. Xi’an, with its rich historical legacy, represents heritage-centered cities where urbanization must integrate conservation. Chengdu, a core city of western China, reflects the trajectory of emerging first-tier cities in Global South. These cities cover diverse regions (e.g., Chengdu and Xi’an in the west, and Shanghai, Nanjing, and Hangzhou in the east), varying population sizes (from 24.87 million in Shanghai to 9.7 million in Nanjing), and different urban hierarchies (Shanghai as a first-tier city, while the others are classified as new first-tier cities). Fig 1 showed more details.

We collected address information from demolition notices across five cities based on published *compensation plans and land acquisition notices* released on municipal and district or county government websites. Data was collected on November 17, 2024, and covered the period from 2021 to 2024. Authors then manually geocoded collected addresses using Gaode Map by converting textual address information into geographic coordinates and constructed geospatial database of demolition sites for subsequent spatial analysis.

Land use and road data were from 30 m annual land cover dataset in 2019 [25], combining with nighttime light remote sensing data and the urban building census database from *Open Street Map* in 2019 (https://www.openstreetmap.org/). Chinese population and Gross Domestic Product (GDP) data in 2020 with 1 km spatial resolution was from *Resource and Environment Science and Data Center* (https://www.resdc.cn/Default.aspx) [26,27] [accessed Jue 7, 2025]. To convert the gridded data into vector-based spatial units, we followed a multi-step processing procedure. First, a regular spatial fishnet was generated to divide the study area into 1 km × 1 km grid cells to ensure consistency with the spatial resolution of raster data. Second, we converted the raster-based population and GDP data into vector format and spatially assigned population values to the corresponding fishnet cells through spatial overlay operations to build a vector-based population and GDP dataset. Finally, to improve the data accuracy and consistency, we calibrated the vectorized population and GDP data using aggregate population counts and GDP from *China’s Seventh National Population Census* and *National Bureau of Statistics of China* at the administrative-unit level. We adjusted total population and GDP within each administrative boundary matched official census figures and preserved the relative spatial distribution of population within each unit.

## Methods

To address the complexity of demolition as a spatially embedded urban process, we adopted a sequential three-stage analytical framework that integrates spatial pattern detection, spatially varying regression analysis, and policy text interpretation. This design allows us to move from identifying macro-scale spatial regularities, to examining place-specific socioeconomic associations, and finally to interpreting institutional and policy contexts that shape demolition practices.

Firstly, we utilized spatial analysis tools, including *Moran’s I* and Kernel Density Estimation (KDE), to visualize and compare the spatial patterns of demolition across the five cities. *Moran’s I* assessed whether demolition sites exhibited statistically significant spatial autocorrelation, while KDE visualized concentration patterns and identified dominant spatial configurations [28,29]. These methods provided a necessary foundation for understanding whether demolition operates as a spatially structured process rather than a random or purely administrative phenomenon.

**Fig 2:**
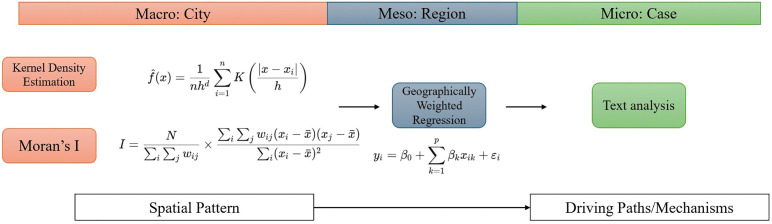
Multi-scale analytical framework for spatial patterns and driving mechanisms.

Then, where spatial autocorrelations were significant, we applied Geographically Weighted Regression (GWR) to examine how the relationships between demolition and selected socioeconomic indicators varied within and across cities. Unlike global regression models that assume spatially stationary relationships, GWR allows regression coefficients to vary locally, thereby capturing spatial heterogeneity across different urban contexts [30,31]. This approach is particularly appropriate given the heterogeneous urban structures and development trajectories of the five cities examined in this study. Finally, we conducted text analysis of policy documents related to demolition and urban redevelopment. This step aims to contextualize and interpret spatial and statistical findings by identifying policy narratives, planning priorities, and institutional logics that inform demolition practices in different cities. Insights from this stage helped synthesize the empirical results into a broader conceptual framework. Fig 2 showed the details of methodology.

## Results

### Spatial pattern

**Fig 3:**
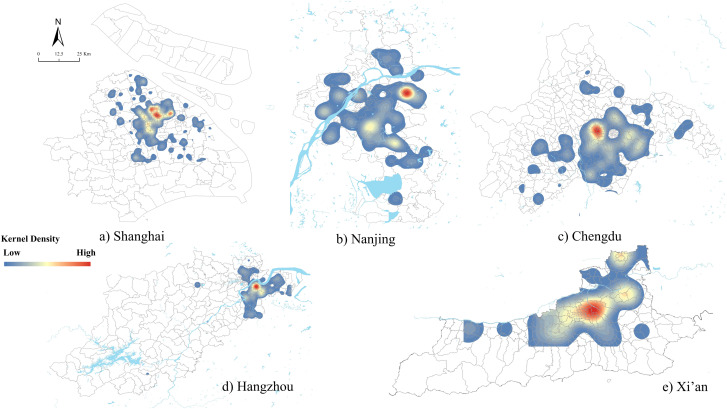
Kernel density estimation results show a “single hot core” pattern in all five cities.

Fig 3 showed the spatial distribution of demolition exhibited a “single hot core” pattern in all five cities. In Shanghai, demolition sites primarily concentrated around Shanghai Railway Station, Shanghai West Railway Station, and along Metro Line 11, predominantly in the northwestern part of the Huangpu River. Most of these sites were previously occupied by industrial enterprises (mainly in the secondary sector), furniture markets, and old residential buildings. Similarly, most demolition projects were in Qingyang District in central Chengdu. Chengdu was in the “residential gentrification” phase, where urban renewal primarily converted older residential areas into commercial or higher-end residential spaces.

Instead of being concentrated in the central business district (CBD) or major transportation hubs, demolition sites were predominantly located in the northern part of Jiangning District in Nanjing, particularly in Tangshan subdistrict, and at the junction between Jiangning District and the main urban area. Many of these sites were previously industrial facilities near Tangshan (Tang Mountain), an area rich in hot spring resources. According to the *National Tourism Resort Master Plan*, the area will be redeveloped to a hot spring resort center. We observed a similar pattern in Hangzhou, though with a distinct development trajectory. Xiaoshan District, designated as Hangzhou’s “charming international gateway”, served as the primary demolition site. This area represented a typical suburban urbanization zone with strong transportation advantages, including proximity to Xiaoshan International Airport, Hangzhou East Railway Station, and Hangzhou South Railway Station. It was also well-connected by Metro Lines 2, 6, and 7, as well as the airport express line and major highways such as the Airport Expressway, Tongcheng Expressway, and Hangzhou-Ningbo Expressway. Most of the demolished sites consisted of former rural villages, with gentrification as the primary redevelopment goal, transforming them into high-end commercial and residential areas.

In Xi’an, demolition projects mainly concentrated in Baqiao District, an area known for its World Expo park and favorable ecological environment. The demolition sites were predominantly rural collective land, earmarked for tourism development and residential infrastructure upgrades. Another sub-hotspot emerged in the northern part of Xi’an, within the Xixian New Area, a national-level new district themed around innovative urban development.

### Driving factors

Based on relevant policy and planning documents, demolition in Chinese cities is closely associated with economic/area development. Guided by established theories in urban geography and political economy, we selected four development-related indicators as potential driving factors of demolition: road network density (defined as the total length of roads per unit area), population density, GDP, and the kernel density of commercial facilities. Road network density reflects infrastructure accessibility and state-led investment, which are central to redevelopment priorities and land-use restructuring [20,32,33]. Population density captures demographic pressure and land competition, which have long been linked to redevelopment and clearance in urban land rent and gentrification theories [34,35]. GDP serves as a proxy for economic capacity and capital accumulation, which reflects the role of local economic growth and fiscal incentives in driving urban restructuring and demolition [36,37]. The density of commercial facilities represents functional centrality and agglomeration economies, which are frequently associated with redevelopment pressure in urban cores [38]. Together, these variables capture key infrastructural, demographic, economic, and functional dimensions that are widely recognized as fundamental drivers of urban redevelopment and demolition processes.

Variance Inflation Factor (VIF) analysis indicated that kernel density of commercial facilities and GDP exhibited multicollinearity. Given their conceptual overlap in representing economic intensity and centrality, we excluded the kernel density of commercial facilities from further analysis.

### Road network density

**Fig 4:**
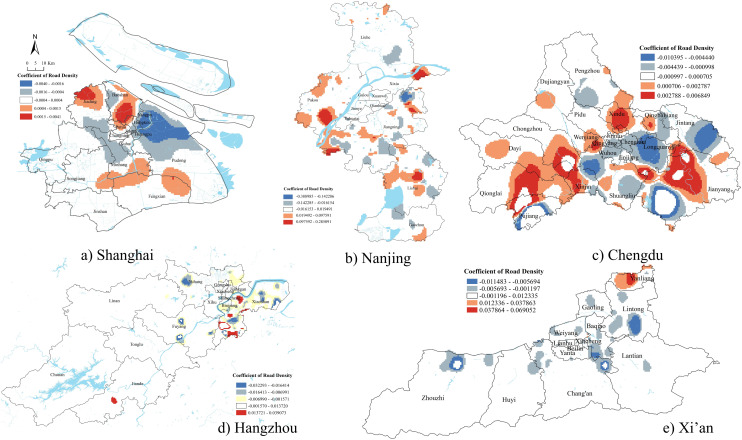
GWR results of road network density.

Road network density had both positive and negative associations with the number of demolition sites across all five cities (Fig 4). In downtown Shanghai, areas with lower levels of transportation experienced more demolition, which suggests that most demolitions occurred in relatively traffic disadvantaged neighborhoods. In peripheral districts such as Jiading and Baoshan, demolition was more likely to occur in areas with higher levels of transportation development. This aligned with the logic of value-oriented land redevelopment [39], where urban land was redeveloped primarily to increase land value, property tax revenue, or real estate profit, often prioritizing economic returns over social equity. Chengdu followed a similar pattern, exhibiting a negative relationship between road network density and demolition in core districts (Wuhou, Qingyang, and Chenghua) and a positive relationship in peripheral areas such as Jianyang, Dayi, and Xindu.

In Hangzhou, Nanjing, and Xi’an, there was no significant relationship between demolition and road network density in downtown areas. These three cities were well known for their historical and cultural heritage, with many protected historical structures in city centers, making demolition more difficult. Nevertheless, each city displayed different demolition patterns in their suburban areas. In Hangzhou, demolition in areas closer to the city center were positively associated with road network density. In Nanjing, there was no clear spatial distribution pattern. In Xi’an, except for the Yanliang area, most demolition occurred in transportation developing zones surrounding the downtown area. This pattern reflected Xi’an’s position as a newly emerging first-tier city undergoing both fast urban renewal and spatial expansion.

### Population density

**Fig 5:**
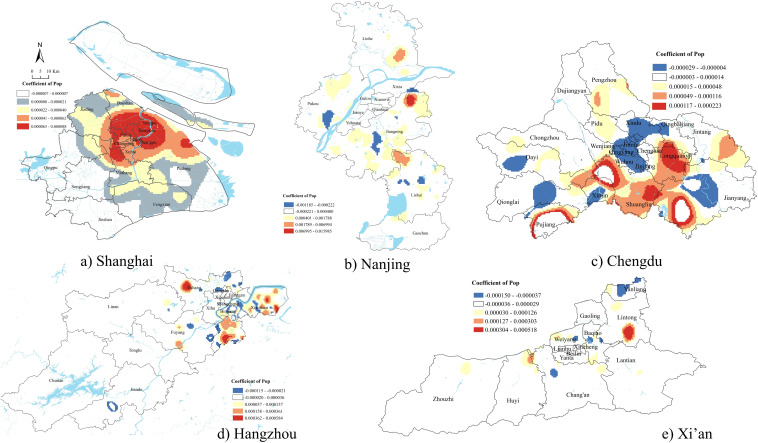
GWR results of population density.

Fig 5 showed most demolition was in densely populated areas in Shanghai. The positive correlation between population density and the number of demolition sites might reflect both the development pressure in densely populated areas and the strategic logic of land redevelopment. High-density zones often carried higher land value potential, which made them prime targets for renewal. These findings aligned with Shanghai’s role as an international and economic metropolis of China.

The population density in other cities had both positive and negative associations with the number of demolition sites. Similar to the findings of road network density, there was no significant relationship between demolition and population density in the downtown areas of Hangzhou, Nanjing, and Xi’an. However, where significant, population density tended to show the opposite pattern of road network density in relation to demolition sites. In Xi’an, demolition was positively associated with road network density but negatively associated with population density in Yanliang, while the opposite pattern was observed in Lintong. This suggested that demolition in these cities might follow dual logic: on one hand, targeting areas with well-developed infrastructure regardless of population concentration to support future growth and connectivity; on the other hand, intervening in densely populated but infrastructurally underdeveloped areas for urban renewal, social upgrading, or relocation-driven spatial rebalancing. We also found this pattern in certain areas of Chengdu, such as Longquanyi.

Unlike other cities, Chengdu exhibited a significant negative relationship between population density and demolition in the city center, with demolition more likely in less densely populated central areas. Such government policies might aim to reduce overcrowding and infrastructure burden in the city core by promoting population dispersion and urban decongestion. Demolition in less densely populated central areas could align with plans to relocate industrial land, low-efficiency housing, or aging infrastructure to create space for parks, public services, or higher-end developments.

### GDP

**Fig 6:**
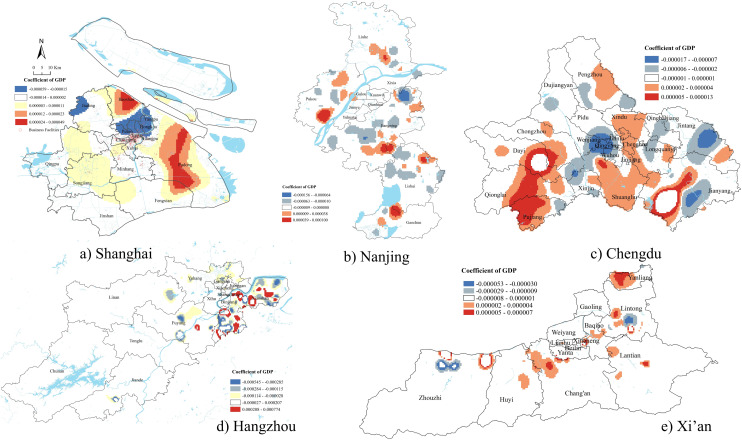
GWR results of GDP.

Fig 6 indicated the demolition number was significantly negatively associated with GDP in the centers of Shanghai and Chengdu. Demolition was more likely to occur in economically disadvantaged central areas, possibly older neighborhoods with aging infrastructure or lower land efficiency. However, in fast-developing areas surrounding the city center, such as Pudong New District in Shanghai and Xindu District in Chengdu, the relationship turned positive. Demolition in these peripheral zones targeted economically strategic areas with high development potential, supporting urban expansion, industrial upgrading, and infrastructure-led growth.

We found a positive relationship between the demolition number and GDP in most areas of Xi’an. This pattern might be explained by the city’s long urban development history, which resulted in a greater presence of older constructions compared to other cities. As a result, demolition activities were more likely to begin in economically valuable areas where land redevelopment could generate higher returns. Demolition served as a tool for maximizing land value through the transformation of historically built-up yet economically strategic zones.

### Land use

**Fig 7:**
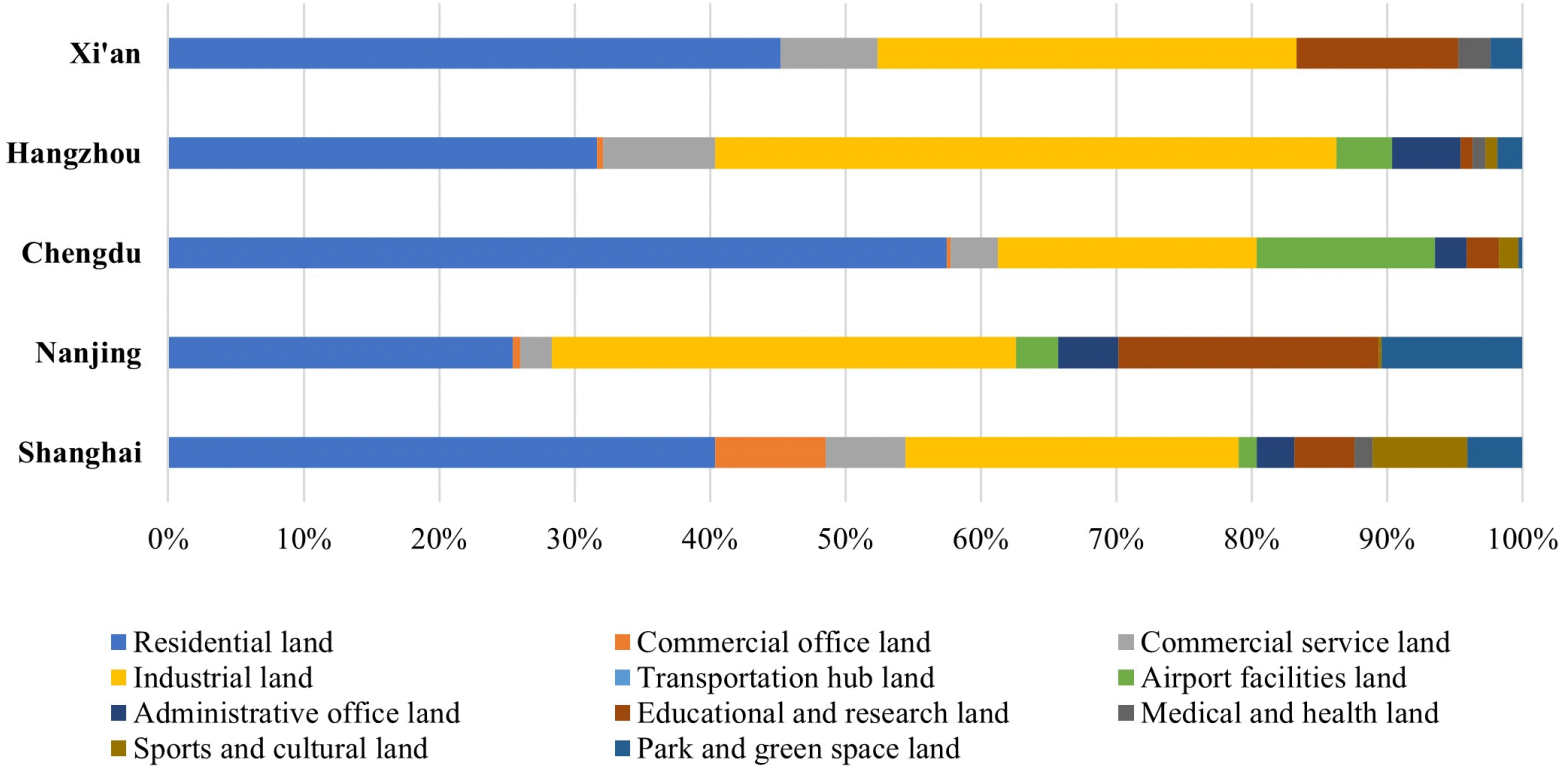
Proportion of different land use categories involved in demolition.

We divided research areas into the smallest land parcels defined by traffic cells, which were delineated using road networks and water systems. Next, we classified each parcel using satellite imagery, nighttime light data, Points of Interest (POI) data, and mobile signaling data. Finally, we categorized land use into 11 urban-specific types: residential land, commercial office land, commercial service land, industrial land, transportation hub land, airport facilities land, administrative office land, educational and research land, medical and health land, sports and cultural land, and park and green space land. Fig 7 showed the proportional distribution of land-use types involved in demolition across the five cities.

Across all cities, demolition activities were disproportionately concentrated in residential and industrial land, which indicates that demolition primarily functions as a mechanism for restructuring everyday living spaces and production-oriented land uses rather than institutional or ecological land. This pattern suggests that demolition is closely aligned with urban growth and redevelopment priorities, particularly where land-use conversion can generate higher economic returns or facilitate functional upgrading. However, the relative importance of specific land-use categories varies systematically across cities, which reflects differences in development stage, economic orientation, and planning objectives.

Shanghai’s demolition concentrated in old residential areas (33.5%) and former industrial zones (20.4%), likely to make room for high-end housing and commercial development. Starting from 2005, the aim of demolition in Shanghai has transformed from urbanization to urban regeneration and gentrification, leading to the “Historic Preservation & Reconstruction Demolition” model. As urbanization in Shanghai matures and its position as an international city solidifies, heritage protection has received increasing attention. However, the tension between heritage preservation and internationalization persists. In contrast, Nanjing exhibited a more diversified demolition profile, with notable shares in residential (11.5%), industrial (15.5%), educational (8.7%), and park and green space land (4.7%). Compared to other cities, Nanjing showed a higher proportion of demolition involving institutional and public land, which suggests a state-led restructuring strategy focused on reallocating land uses to support industrial upgrading and administrative consolidation. Examples included consolidating universities and allocating parkland for infrastructure (Industrial Park Upgrading model). Nanjing’s tourism industry is often considered misaligned with its reputation for rich heritage, as relatively few people are aware of its cultural assets. “Industrial Heritage Revitalization” will be the goal for Nanjing. This pattern highlights demolition as a planning instrument for reorganizing public functions rather than solely facilitating real estate development.

Hangzhou owned the largest demolition area and the highest share of demolition in industrial land (35.8%). This concentration reflects Hangzhou’s role as a digital economy hub, where industrial land is actively converted into technology campuses, business districts, and commercial real estate. Given the rapid development of the tertiary sector, infrastructure construction appears to lag. Limited housing cannot accommodate the rapidly growing population attracted by increasing job opportunities. Consequently, “Urban Village Redevelopment” has become the leading model of its demolition, with “Migrant Population Relocation” as the primary objective. Urban redevelopment in Xi’an focused on residential upgrading in older neighborhoods, but less emphasis on commercial or institutional relocation. This pattern suggests a more conservative redevelopment approach, shaped by heritage preservation constraints and relatively lower market-driven redevelopment pressure. Chengdu demonstrated a distinctive profile characterized by a higher proportion of demolition related to park and green space integration. This reflects the city’s “Park City” development philosophy, where demolition is strategically employed to support ecological restoration and spatial rebalancing rather than intensive land commercialization.

Fig 7 and Table 1 revealed that demolition is not randomly distributed across land-use categories but operates as a strategic tool of urban land-use restructuring. While residential and industrial land consistently form the primary targets of demolition, variations in land-use composition across cities reflect differing economic roles, planning priorities, and stages of urban development. These findings underscore demolition as a spatially differentiated policy instrument through which cities reorganize land functions, manage growth pressures, and negotiate trade-offs between development, heritage, and social equity.

**Table 1:** Overview of demolition projects in five cities (2000–2025).

City	Time Span	Demolition Sites (No.)	Affected Area (km²)	Dominant Renewal Type	Key Conflicts/ Features
**Shanghai**	2005–2025	1,240	58.7	Historic Preservation & Reconstruction	Heritage vs. Internationalization
**Nanjing**	2003–2025	920	42.3	Industrial Park Upgrading	Industrial Heritage Revitalization
**Hangzhou**	2010–2025	1,580	67.9	Urban Village Redevelopment	Migrant Population Relocation
**Xi’an**	2008–2025	1,360	61.2	Heritage Site Renewal	Conservation vs. Development Balance
**Chengdu**	2007–2025	1,050	39.8	Park City Integration	Ecology-Development Coordination

### Typology of demolition

Drawing from the results above, we identified three major types of demolition based on the dominant spatial and development-related paths: urban center pattern, nearby suburb pattern, and dual-zone demolition pattern (Table 2).

**Table 2:** Typology of demolition.

Demolition Pattern		Spatial Focus	Key Socioeconomic Characteristics	Dominant Land-Use Change	Representative Cities	Planning Logic
**Urban Center Pattern**		Traditional city core	- High population density; - Relatively low GDP performance in specific inner-city zones; - High land value pressure	Low-efficiency residential and industrial land ➔ high-end residential and commercial uses	Shanghai (Putuo and Jing’an); Chengdu	Government-led inner-city renewal and gentrification
**Nearby Suburb Pattern**	**Transformation-led**	Urban fringe/ peri-urban zones	- Low population density; - Moderate to high GDP growth potential; - Underutilized collective or industrial land	Agricultural or industrial land ➔ tourism, commercial, or mixed-use functions	Nanjing (Tangshan); Xi’an	Strategic land-function conversion aligned with regional economic restructuring and policy planning
	**Updating-led**	Established suburban neighborhoods	- Moderate to high road network density; - Varied GDP and population density	Functional continuity with substantial increases in density, land value, and built-environment quality	Hangzhou (Xiaoshan)	Infrastructure-driven suburban modernization
**Dual-Zone Demolition Pattern**		Inner city and peripheral zones	- Inner city: lower GDP and road density; - Suburbs: higher GDP and road density	Inner-city renewal combined with suburban industrial expansion and infrastructure development	Shanghai (Jiading and Baoshan); Chengdu (Qingyang, Jianyang, and Xindu)	Two-tiered strategy balancing densification and territorial expansion

### Urban center pattern

The urban center pattern referred to demolition activities concentrated in the traditional city core, primarily aimed at revitalizing high-density areas with outdated infrastructure and low land-use efficiency. High population density and land value pressures shaped this pattern, as well as relatively low GDP performance in specific inner-city zones. This indicated a government-led effort to renew economically underperforming yet strategically located areas. Shanghai and Chengdu were representative of this pattern.

### Nearby suburb pattern

We further divided nearby suburb pattern into transformation-led and updating-led. Peripheral zones of cities such as Nanjing and Xi’an represented transformation-led demolition patterns. These areas often consisted of collective land, industrial facilities, or rural-urban fringe zones that lacked integration into the urban fabric. The key drivers were strategic policy planning, availability of underutilized land, and alignment with regional economic restructuring objectives. These areas typically exhibited low population density and moderate to high GDP growth potential, making them ideal candidates for land function conversion.

The updating-led pattern focused on renewing the existing functions of suburban neighborhoods through demolition and rebuilding, without drastically altering their land use category. GWR results suggested that road network density was positively correlated with demolition, while population density showed no clear pattern, which indicates that infrastructure accessibility, rather than social need, guided demolition decisions. The strategy reflected local government efforts to modernize suburban environments while enhancing urban competitiveness.

### Dual-zone demolition pattern

The dual-zone demolition pattern combined simultaneous demolition efforts in both the urban core and peripheral zones, supporting a dual logic of inner-city renewal and suburban expansion. GWR results showed negative correlations with GDP and road density in the city core, and positive correlations in the suburbs. This hybrid approach highlighted how demolition policy could serve both densification and territorial expansion goals within a single metropolitan framework.

## Discussion

Demolition is not only a challenge in Global South but a global urban issue intersecting economics, sociology, urban geography, and law. In New York City, a private developer has proposed demolishing multiple public housing complexes, including a senior building housing 91 older adults, despite strong community opposition [40]. By analyzing demolition projects across five cities in Global South, this study offers broader insights into global urban planning and development under the rapid urbanization and gentrification.

### Multidimensional impacts of urban demolition

#### Social dimensions.

Consistent with existing research on the unequal impacts of natural disasters [41], pandemics [42–44], and displacement [45,46], low-income households have been disproportionately affected. More than 60% of original tenants in cases such as Shanghai’s Ruikangli and Hangzhou’s Wangjiangmenwai relocated to peripheral areas, which leads to a 35% decline in access to public services [47].

Community networks, meanwhile, display divergent trajectories: in Xi’an’s Huifang redevelopment, dispersal of the Muslim population reduced religious participation by 52%, exemplifying a “fracture” model [48], whereas Chengdu’s Caojiaxiang project preserved 87% of neighborhood ties through a resident-led renewal alliance, thereby enhancing resilience [49].

#### Environmental dimensions.

Demolition produces notable environmental repercussions and intensifies urban heat island effects as land surface temperatures rose by 2.8–4.3 °C within three years, driven by vegetation loss, higher building density, and increased asphalt coverage [50]. Studies in Bangkok indicated that urbanization can increase land surface temperatures by 4.8°C [51].

Demolition drove this process through three key mechanisms: the fragmentation of green space, with vegetation cover declining by more than 40% in Hangzhou [52]; the sharp increase in building density, with floor area ratio rising from 1.2 to 2.8 in Shanghai’s Jinling East Road [53]; and material albedo change, with asphalt coverage expanding by 32 percentage points [54]. Equally important, the cooling radius of redeveloped plots has declined significantly, with their capacity to reduce surrounding temperatures within 100 meters dropping by 56%, thereby intensifying heat exposure risks [55]. Together, these processes not only amplify local heat stress but also exacerbate environmental inequities, with Xi’an data showing that low-income resettlement zones face heat risk levels 3.2 times higher than affluent neighborhoods, a phenomenon aptly described as “climate apartheid.” [56–58]

#### Economic-culture dimensions.

Economically, demolition triggers industry’s “genetic mutation” [59,60]. There are three distinct models: a gentrification-led model, as in Shanghai’s Yuyuan Road and Nanjing’s Xiaoxihu, where local services declined by 68% and residents’ costs rose [61,62]; a hybrid coexistence model, exemplified by Chengdu’s Yulin East Road, where adopted the “golden corners and silver edges” strategy, retaining 45% of the original shops while adding cultural and creative bookstores and community cafés, thereby forming a symbiotic network of old and new business formats [63,64]; and an industrial upgrading model, such as Wuxi’s Lanxi Industrial Park, which adopted a “rent + property + profit-sharing” model to facilitate the transformation from traditional manufacturing to a creative design cluster.

Cultural identity is another dimension profoundly affected by demolition, particularly in historic urban neighborhoods where everyday spaces serve as repositories of collective memory and intangible heritage. In Nanjing’s Menxi district, for example, the demolition process led to a 70% reduction in spaces associated with intangible cultural practices, including small workshops, informal gathering places, and neighborhood-based cultural venues, thereby disrupting mechanisms of local knowledge transmission and intergenerational continuity [65].

#### Urban demolition complex systems.

**Fig 8:**
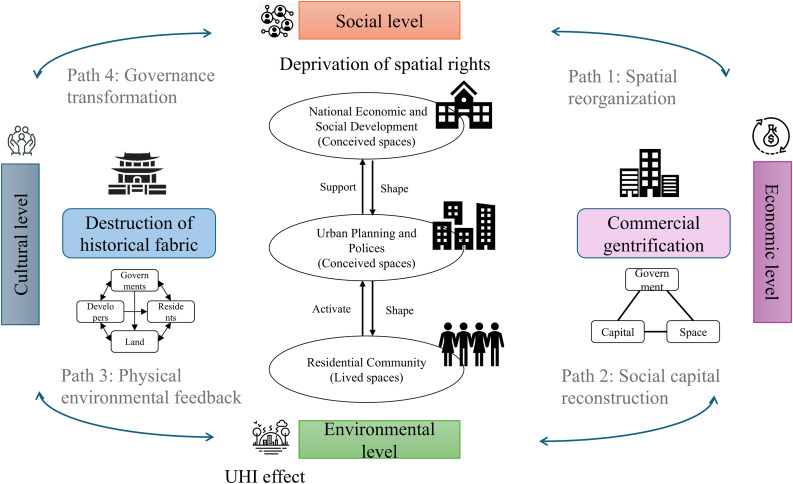
Urban demolition complex systems.

Demolition simultaneously reshapes social networks, environmental conditions, and economic landscapes, which highlights the complex trade-offs and equity concerns inherent in contemporary urban redevelopment. Existing research has mostly focused on single-dimensional impacts, lacking analysis of the systemic “spatial-social-environmental” interactions, and even fewer studies have established comparative frameworks based on large-sample, multi-city data. To fill the gap, we proposed “Urban Demolition Complex Systems” (UDCS) theory (Fig 8).

UDCS implied changing inter-relationship between the government, capital, and space. Most researchers focused on how the state and the market worked together under neoliberalism [66]. Others looked more at how social life was shaped by space and how different scales, local, national, global, were connected in complex ways [67,68]. China has special political system and land use policy. On one hand, many believed the Chinese state has played a key role in driving the country’s rapid urban changes since the market reforms of the 1980s [69,70]. On the other hand, the state was also criticized for creating institutional barriers, such as the *hukou* system, that limited labor market flexibility, restricted people’s movement, reduced economic efficiency, and contributed to social inequality [71,72]. Demolition was a typical government-led example to analyze both perspectives. We borrowed Henri Lefebvre’s theory to explore how state-led space was imagined, experienced, and challenged by people with different roles and interests [73]. Unlike the triangle models proposed by Chao [73], the demolition process tended to follow a top-down approach to spatial restructuring. From Lefebvre’s theory of the production of space, demolition was a socio-spatial practice that reflected how government power materialized in urban space. The government produced space based on the overall strategic plan (i.e., 14^th^ Five-Year Plan for National Economic and Social Development of the People’s Republic of China) (conceived space), framed it through district/city-level policy and propaganda (perceived space), and ultimately reshaped how people experienced and inhabited their environments (lived space). Demolition often disrupted the lived space most dramatically and generated social protest and emotional tension, especially when it conflicted with higher-level space (i.e., conceived space and perceived space). Conflict theory offered a critical lens to understand structural inequalities and spatial power struggles. According to conflict theory, social change was driven by tensions and confrontations between groups with unequal access to resources and power [74]. In the context of demolition, the government and developers often represented dominant forces, driving spatial restructuring through policies, planning, and capital, while original community residents were typically marginalized. These residents faced the loss of housing, identity, and cultural space, which frequently led to various forms of resistance. Yet, the *hukou* system further prevented displaced rural migrants or informal residents from accessing urban social services or resettlement opportunities. While the government promoted demolition as a form of modernization, the lived realities of displaced residents, especially those without urban *hukou*, revealed a spatial logic of exclusion, dislocation, and contestation.

Chinese cities shared some common disputes, including conflicts over resettlement housing quality, historical property rights, and lack of transparency, which reflected deeper structural roots such as unequal benefit distribution and information asymmetry. To solve these problems, Hangzhou’s “Sunshine demolition” model emphasized full procedural transparency, public participation, and online disclosure of compensation standards, which effectively reduces conflicts; Chengdu’s community mediation committees integrated local knowledge and negotiation practices to address disputes at the grassroots level before they escalated. Beyond immediate conflicts, another layer of tension arose between urban renewal and cultural preservation. In historically rich neighborhoods, such as Beijing’s hutongs or Fuzhou’s Three Lanes and Seven Alleys, demolition could threaten intangible heritage and collective memory. Cities responded with varied strategies: Suzhou’s “micro-renewal” approach avoided wholesale demolition, instead promoting incremental, community-sensitive improvements to retain historical character while enhancing livability. These examples showed that urban space was produced not only by state power but also by negotiation, contestation, and adaptation. Integrating conflict theory and Lefebvre’s spatial triad enabled us to analyze how state-led demolition was both resisted and redefined, transforming demolition into a dynamic process of socio-spatial reconfiguration, where embedded with questions of justice, memory, and belonging.

### Limitations

Although this research has revealed various driving paths of demolition, there are still several limitations. First, while the selected cities represented different strategic urban roles, they were (emerging) first-tier cities with relatively high levels of economic development. Future studies should expand the scope to include second- and third-tier cities, as well as rural areas, to capture more diverse urban-rural dynamics and spatial variations in the demolition process. Secondly, future research can incorporate multi-source data, including corporate demolition project reports, resident interviews or online comments, and spatial data.

Thirdly, further studies can explore national and local policy like compensation standards, evaluation methods, and implementation outcomes. In first-tier cities where land value is high, monetary compensation may be preferred, whereas in second-tier cities, in-kind resettlement (i.e., physical relocation) might be more common. Fourthly, future research can investigate how demolition reshapes urban space and reinforces social stratification through qualitative methods. Interviews can be used to examine the migration trajectories of displaced low-income residents, their integration into new communities, and how spatial fragmentation may exacerbate class solidification. We can also apply mixed methods, using big data to track population mobility and employment shifts, combined with surveys to capture processes of social network disruption and reconstruction. Finally, while this study focuses on identifying the spatial patterns and driving mechanisms of demolition, future research could examine how demolition sites might be leveraged as strategic opportunities to support more multicentric and spatially balanced urban development.

## Conclusion

Demolition is a complex social phenomenon involving multiple stakeholders, yet research from a macro-level geographical perspective remains limited. This study addressed that gap by applying spatial analysis tools to big data from five representative Chinese cities with various developing paths. We found, in all five cities, the spatial distribution of demolition exhibited a “single hot core” pattern. Demolition was not driven by a single factor but rather by a complex interplay of urban infrastructure, population distribution, and economic priorities. Road network density and population density had varied influences depending on location, which suggests that demolition served different spatial logics: either upgrading high-density, underdeveloped neighborhoods or preparing well-connected areas for future development. GDP’s role as a driver further emphasized this dual logic, while economically weaker central areas were subject to clearance due to low land efficiency, economically strategic outer zones were also prioritized for demolition to facilitate industrial upgrading and urban growth. There were three patterns of demolition: the Urban Center Pattern (e.g., Shanghai, Chengdu) focused on renewing dense inner-city areas; the Nearby Suburb Pattern included transformation-led redevelopment of fringe zones (e.g., Nanjing, Xi’an) and updating-led upgrades of suburban neighborhoods (e.g., Hangzhou); and the Dual-Zone Pattern combined both inner-city renewal and suburban expansion to support balanced urban growth.

This study contributes theoretically by demonstrating that demolition is not merely a planning instrument, but a socio-spatial restructuring process shaped by uneven development dynamics and multi-scalar governance priorities with the innovative UDCS framework. Understanding the demolition mechanism could help unpack the socio-spatial logic of global urban transformation and offer policy implications for urban governance and redevelopment planning. The findings suggest that policymakers should move beyond growth-oriented and efficiency-driven redevelopment models. Given that demolition tends to concentrate in spatial “hot cores” where infrastructure, capital, and development pressure intersect, targeted safeguards are needed to prevent the disproportionate displacement of vulnerable populations, particularly residents constrained by institutional barriers such as the hukou system. Transparent compensation mechanisms, participatory planning processes, and community-level mediation platforms may help reduce conflict and enhance procedural justice. Future research could move beyond macro-structural analysis to examine how demolition reshapes lived experiences and place attachment at the neighborhood level. Large-scale clearance and redevelopment disrupt long-established social networks, everyday routines, and emotional bonds to place. Integrating macro-geographical restructuring with micro-level place attachment dynamics, through qualitative or mixed-method approaches, would provide a more comprehensive understanding of the social consequences of demolition.
